# Exercise Boosts the Immune System and Enhances Immunotherapy Responses in Pancreatic Cancer and Mesothelioma

**DOI:** 10.3390/biom16040493

**Published:** 2026-03-25

**Authors:** Brindley Hapuarachi, Sarah Danson, Jonathan Wadsley, Hannah Brown, Phoebe Southam, Munitta Muthana

**Affiliations:** 1 Division of Clinical Medicine, University of Sheffield, Sheffield S10 2TN, UK; s.danson@sheffield.ac.uk (S.D.); hannahbrown1306@gmail.com (H.B.); psoutham1@sheffield.ac.uk (P.S.); 2 Sheffield Teaching Hospitals NHS Foundation Trust, Sheffield S10 2JF, UK; jonathan.wadsley1@nhs.net

**Keywords:** exercise, immunotherapy, immunology, tumour microenvironment

## Abstract

Background: Exercise modulates the immune system and may enhance anti-cancer activity, offering potential synergy with cancer immunotherapy. Tumours with low immune cell infiltration (“cold” tumours) often respond poorly to immunotherapy and are associated with poor prognosis. Here, we demonstrate that exercise can reshape the immune landscape of tumours across the cold spectrum. Methods: *C57BL/6* mice underwent orthotopic implantation of PANC02 (murine pancreatic adenocarcinoma) cells and *BALB/c* mice underwent intraperitoneal injections of AB-1 (murine mesothelioma) cells. Mice were then divided into groups; exercise with anti-Programmed Cell Death Protein 1 (PD-1), exercise with isotype, no exercise with anti-PD-1 and no exercise with isotype. Treadmill-running was performed for 20 min/day, 4 days/week at a speed of 12 metres/minute. Resistance training consisted of hanging upside down on a wire-mesh screen for 1 min 2 days/week. Flow cytometry was used to measure TME immune populations. Tumour and liver samples were harvested, paraffin wax-embedded/sectioned and analysed using SlideViewer 2.9.0™. A total of 22 healthy volunteers underwent a single bout of high-intensity interval cycling. Blood was collected pre- and post-exercise. Flow cytometry was used to measure leucocyte subpopulations. MSTO-211H (mesothelioma) and PANC-1 (pancreatic cancer) cells were cultured with pre- and post-exercise serum, with/without HSV1716, and viability determined using alamarBlue^®^. PANC-1 apoptosis and migration were assessed using caspase-3/7 and scratch assays, respectively. Results: In an orthotopic pancreatic cancer mouse model, combining exercise with immunotherapy significantly increased tumour necrosis and reduced metastatic potential. In both pancreatic cancer and mesothelioma models, this combination remodelled the tumour microenvironment, enhancing cytotoxic CD8^+^ T cell infiltration, upregulating Programmed Cell Death Protein 1 (PD-1), and reducing Myeloid-Derived Suppressor Cells and regulatory T cells (Tregs). Complementary human studies revealed an acute systemic release of Natural Killer cells and a reduction in Tregs following high-intensity interval exercise in healthy volunteers. Moreover, exercise-conditioned serum from these participants exerted anti-cancer effects on pancreatic cancer and mesothelioma cell lines. Conclusions: Altogether, these findings highlight exercise as a promising adjunct to immunotherapy for poorly immunogenic cancers such as pancreatic cancer and mesothelioma.

## 1. Introduction

Aerobic exercise has known benefits for physical and mental health in both the general population and in patients with cancer. In cancers such as breast and colorectal cancer, exercise has been shown to reduce the risk of cancer recurrence following curative surgery and improve overall survival [1,2]. The National Health Service (NHS) and World Cancer Research Fund (WCRF) guidelines recommend 150 min of moderate-intensity or 75 min of high-intensity exercise per week [3,4]. However, this is not specifically tailored to the cancer population and is the basis for general health benefits. To understand how best to integrate exercise prescription into oncology clinical practice, a clearer demonstration of the potential anti-tumour effects propagated by exercise in different cancers is required. Exercise-induced leucocytosis, myokine release and tumour vasculature normalisation are all proposed mechanisms behind the observed tumour cytotoxicity and highlights the potential of transforming ‘cold’ immune-deplete tumours to ‘hot’ immune-enriched tumours [5]. Consequentially, tumours would be more susceptible to anti-cancer therapies including immunotherapy.

There is a clear unmet need to advance treatment options and survival outcomes in poor-prognostic cancers that lie at the ‘cold’ end of the tumour microenvironment (TME) spectrum. These include pancreatic cancer and mesothelioma, with 5-year survival rates of less than 7% and 12%, respectively [6,7]. Incorporating exercise alongside the treatment pathway for these cancers may allow for intratumoural immune cell infiltration, enhancing the immune attack on tumours and supporting other anti-cancer therapies.

Due to the complex interactions between exercise and the immune system, mouse models were used to show that aerobic exercise and resistance training alongside immune checkpoint inhibitors can modulate the pancreatic cancer TME, increasing tumour responses and reducing metastatic disease. This study also characterised the immediate systemic immune response to a high-intensity interval aerobic exercise activity in healthy volunteers. We also demonstrate the anti-cancer effects of exercise-conditioned serum on pancreatic cancer and mesothelioma cells in vitro.

## 2. Materials and Methods

### 2.1. Cell Lines and Culture

Mesothelioma cells (MSTO-211H) were obtained from collaborators Dr Sarah Haywood Small and Mr Sam Bonsall at Sheffield Hallam University. MSTO-211H cells were obtained from the pleural effusion of a 62-year-old white male with biphasic mesothelioma (MSTO-211H–CRL-2081|ATCC, VA, USA) [8]. The MSTO-211H cells were incubated in complete Roswell Park Memorial Institute (RPMI) 1640 medium (GIBCO, Germany) with 10% foetal bovine serum (FBS), 1% penicillin–streptomycin and 1% fungizone.

Pancreatic cancer cells (PANC-1) were provided by collaborators Dr Helen Matthews and Dr Victoria Hart at the University of Sheffield. PANC-1 cells were obtained from the pancreatic duct of a 56-year-old white male with pancreatic epithelioid carcinoma (PANC-1–CRL-1469|ATCC, Virginia, USA) [9]. The PANC-1 cells were incubated in complete Dulbecco’s Modified Eagle Medium (DMEM; GIBCO, Germany) with 10% FBS, 1% penicillin–streptomycin and 1% fungizone.

Murine-luciferase-expressing PANC02 (PANC02-luc) cells (gift from Dr Ameera Jalini, University of Sheffield, UK) were cultured in DMEM with 10% FBS, 1% penicillin/streptomycin and 1% fungizone. PANC02 cells were derived from *C57BL/6* mice.

Mesothelioma mouse-luciferase-expressing AB-1 (AB-1-luc) cells were obtained from Professor Paola Allavena at the Clinical and Research Institute Humanitas, Rozzano (Milano, Italy). The AB-1 cells were cultured in RPMI 1640 with 10% FBS and 1% penicillin–streptomycin. AB-1 cells were derived from *BALB/c* mice.

The cells were incubated within a SANYO incubator set at 37 °C and 5% carbon dioxide (CO_2_) in T75 flasks and used within 30 passages and regularly tested for mycoplasma.

### 2.2. In Vivo Studies

Animal procedures were performed in accordance with the UK Animals (Scientific Procedures) Act 1986 and carried out under a UK Home Office-approved amendment to PP1099883. Six–eight-week-old male and female *C57BL/6* and *BALB/c* mice were purchased from Charles River Laboratory and underwent a 7-day acclimatisation period in the Biological Services Unit, University of Sheffield, prior to any intervention. Group sample size was determined using the NC3Rs Power Calculator (NC3Rs, London, UK). To detect a 5% difference within immune markers between groups with a predicted within-group variability of 2%, a power of 90% and a significance level of 0.05, 5–6 mice per group were required. The primary aims included:

(1) Assessing the effects of treadmill running and resistance training in orthotopic pancreatic cancer and mesothelioma mouse models;
(2) Assessing the impact of exercise and exercise/anti-PD1 therapy on pancreatic cancer and mesothelioma TMEs in mouse models;
(3) Assessing the effect of exercise and exercise/anti-PD1 therapy on tumour necrosis and metastatic disease.

The animals were randomly divided into experimental groups, ensuring that each animal had an equal chance of receiving any treatment. This approach reduces bias, balances baseline characteristics (such as weight or age), and increases experimental reproducibility.

### 2.3. Orthotopic Pancreatic Cancer and Mesothelioma Mouse Models

*C57BL/6* mice (n = 20) received oral meloxicam (5 mg/kg) and were anaesthetised with 5% isoflurane. A 2 × 2 cm area over the left thoraco-abdominal region was shaved. Buprenorphine (0.05 mg/kg, subcutaneous (s.c.)) was administered, and the mice were placed on a 37 °C heating pad. The skin was prepped with 10% povidone iodine. Under 3% isoflurane, 4 × 10^5^ PANC02-luc cells in 20 µL Matrigel/PBS were injected into the pancreatic tail, exposed via gentle spleen retraction. The pancreas and spleen were returned to the cavity, and the incision was closed with 5–0 absorbable sutures (muscle) and 6–0 non-absorbable sutures (skin). The mice were allowed to recover on a 37 °C heated pad. *BALB/c* mice (n = 24) were injected via the intraperitoneal (i.p.) route with 1 × 10^5^ AB-1-luc cells. The mice were monitored for 1–2 h post-injection and throughout for abdominal distension due to malignant ascites. The mice were humanely euthanized when distension exceeded normal limits (comparable to day 11 pregnancy). Tumour growth in both models was monitored weekly using an In Vivo Imaging System (IVIS) following intraperitoneal luciferin injection and 3% isoflurane anaesthesia.

### 2.4. Treadmill Running

Mice in the treadmill group performed moderate-intensity running for 15–20 min/day at a speed of 12 m/min for a minimum of 4 days per week using a modified mouse treadmill (Panlab, Barcelona, Spain) with stimulation by paint brush spike. A modified moderate-intensity regime was used to enhance translatability and reflect the likely capabilities of pancreatic cancer and mesothelioma patients.

### 2.5. Horizontal Screen

For the horizontal screen resistance training, the mouse was placed on top of a 29 × 29 cm wired screen. A stop watch was started, and the screen was inverted. The mouse would be kept upside down, 30 cm over a soft surface, for 1 min. If unable to hang upside down, the time taken for the mouse to fall off the screen was documented.

**Figure 1:**
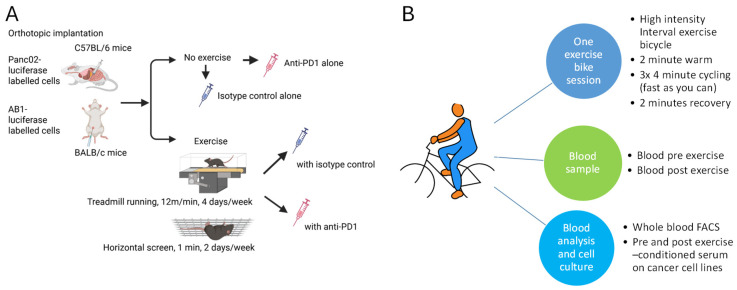
(**A**,**B**) Methodology for mouse and healthy volunteer studies. (**A**) Pancreatic cancer cells (Panc02-luciferase) and mesothelioma cells (AB1-luciferase) were orthotopically implanted into the pancreas of *C57BL/6* mice (via surgery) and *BALB/c* mice (via intraperitoneal injection), respectively. The diagram outlines the different intervention arms. (**B**) Recruited healthy volunteers underwent 2 min of gentle cycling, followed by 4 min of cycling at maximum intensity and then 2 min of gentle cycling or rest. The 4 min high-intensity cycling was then repeated two more times. Intensity was judged subjectively using the Borg scale, which ranges from 6 to 20, to assess Rate of Perceived Exertion (RPE). A target score of 16–20 was used to ensure that the participants were performing at a high intensity. Blood samples taken before and after exercise were immune-profiled and cultured with pancreatic cancer and mesothelioma cell lines to assess exercise-induced changes.

The pilot study consisted of 12 mice in 4 groups (n = 3). All mice underwent orthotopic pancreatic cancer implantation on day 0. Treatment included a sedentary control group and different exercise activities (as per Supplemental Materials Table S2A). The larger pancreatic cancer study included 20 mice in 4 groups (n = 5). The mesothelioma study included 24 mice in 4 groups (n = 6). Exercising mice underwent a combination of treadmill-running and resistance training and were given either 200 ug of anti-mouse anti-Programmed Cell Death Protein 1 (PD1) or 200 ug of rat IgG2a (Selleckchem, Essex, UK) via the i.p. route, as illustrated in Figure 1A and detailed in Table S2B,C (Supplemental Materials).

### 2.6. Exercise Activity

Healthy volunteers (≥18 years) with no significant medical conditions and deemed not to have any risks associated with high-intensity interval aerobic exercise on an exercise bicycle were recruited. All participants provided written informed consent. Ethical approval was obtained from the University of Sheffield in January 2023 (reference number 050457). The participants underwent a single high-intensity interval aerobic exercise session on an exercise bicycle, as shown in Figure 1B.

Blood samples were taken prior to and immediately after the exercise session. Ethylenediaminetetraacetic acid (EDTA) blood tubes were placed on ice whilst red-top tubes were kept at room temperature, allowing the blood to coagulate. Whole blood from the EDTA tubes was prepared for flow cytometry within 60 min. Blood from tubes without anti-coagulant were centrifuged at 2000× *g* revolutions per minute (RPM) for 10 min at 4 °C. The serum was separated, aliquoted and stored at −20 °C. Each aliquot only underwent one freeze–thaw cycle and the samples were analysed within 6 months.

### 2.7. Sample Size

A sample size calculator [10] was used to determine the number of participants required for this study. It calculated that 18 participants were required to demonstrate a 4% increase in immune cells within exercise-conditioned blood with an alpha level of 0.05 at 80% power.

### 2.8. Flow Cytometry

A 200 µL volume of blood taken before and after exercise from each participant was incubated with all 10 optimal antibody–fluorophore conjugate concentrations and Zombie NIR dye (see Supplemental Material Table S1) on ice in the dark for 20 min. Following fixing in 1% paraformaldehyde (PFA), the cells were washed and resuspended in phosphate-buffered saline (PBS), kept in a 4 °C fridge overnight and analysed the next day using a Cytek Aurora Flow Cytometer (CA, USA). Changes in cell counts for each leucocyte population with exercise were analysed using GraphPad Prism 8.0.2.

### 2.9. Migration Assays

For all in vitro assays, 3 independent biological repeats were analysed. PANC-1 cells (5 × 10^5^/well) were seeded in 48-well plates and incubated for 24 h to allow them to reach 100% confluence. Proliferation was inhibited using 12 µg of mitomycin C in serum-free medium for 90 min at 37 °C. A midline scratch was made with a 200 µL pipette tip, followed by treatment with DMEM containing either 10% FBS or 10% pre-/post-exercise-conditioned serum. Scratch images were captured over 0–50 h using an EVOS™ FL Auto Imaging System (ThermoFisher Scientific, MA, USA). MSTO-211H cells (1 × 10^5^ in 100 µL of RPMI) were seeded onto 3.0 µm pore inserts in a 24-well plate. After a 10 min incubation at 37 °C, the wells were filled with RPMI containing either 10% FBS or 10% pre-/post-exercise-conditioned serum. After 2 h, the inserts were removed, and non-migrated cells were cleared. The migrated cells were fixed (4% PFA), stained (0.2% crystal violet), and imaged using an EVOS™ FL Auto. Images were analysed and cells were counted using ImageJ 1.52g (NIH, MD, USA).

### 2.10. Apoptotic Assays

To determine the effect of exercise-conditioned serum on cancer cell apoptosis, PANC-1 and MSTO-211H cells in pre- and post-exercise-conditioned sera were treated with CellEvent™ Caspase -3/7 Red Detection Reagent. A total of 5 × 10^5^ cells per well were seeded onto a 96-well plate and incubated for 24 h until they were confluent. The medium was discarded and then the cells were treated with DMEM and 10% FBS or 10% pre- or post-exercise-conditioned serum and incubated for a further 24 h. A 10 µL volume of the 10X CellEvent™ Caspase -3/7 Red Detection Reagent solution was then added to each well. Following 60 min of incubation at 37 °C, fluorescence was read using an Ensight Multimode Microplate reader (PerkinElmer, CT, USA).

### 2.11. Flow Cytometric Analysis of Tumours

Flow cytometry was used to characterise and quantify the immune cell populations of the pancreatic TMEs. Tumours were harvested at the end of the study and manually dissociated into single-cell solutions, which were stained with primary antibodies to analyse the following immune populations: T-helper (CD45+CD3+CD4+), and regulatory T cells (Tregs) (CD45+CD3+CD4+CD25+), cytotoxic T cells (CD45+CD3+CD8+), Myeloid-Derived Suppressor Cells (MDSCs) (CD45+Ly6C+CD11b+) and Natural Killer (NK) cells (CD45+NK1.1+). Zombie NIR dye was used to detect live/dead cells.

### 2.12. Histology

Pancreatic tumours, along with spleens, livers and lungs, were harvested at the end of the study and placed in 4% PFA for 24–48 h. These tissues were then transferred into 70% ethanol to be sectioned and embedded in paraffin wax prior to haematoxylin and eosin (H&E) staining. H&E slides were scanned and viewed on a SlideViewer^®^. Metastases from liver sections were counted and the percentage area of liver affected by metastases was also measured. Tumour histology was reviewed, and the percentage area of necrosis was measured. Tissue slides were evaluated by researchers without knowledge of the treatment groups, reducing subjective bias.

Paraffin wax-embedded slides stored at room temperature were de-waxed using xylene and then washed with absolute alcohol and hydrated with 90% and then 70% alcohol prior to washing in distilled water. Following fixation in acetone and blocking with 5% goat serum and 10% murine FcR blocking solution, tumours were stained using conjugated antibodies (PE-PD1, FITC-CD8, AF647-MRC1, PE-F4/80 and DAPI (nucleus stain)). A Zeiss LSM 980 Airyscan confocal microscope (Zeiss, Germany) was utilised to visualise the slides.

### 2.13. Statistical Analyses

GraphPad Prism v.8 was used to analyse statistically significant differences and generate graphs. Data are expressed as median values with standard deviations. Due to the sample sizes, non-parametric tests were used to analyse the data unless the data was confirmed to follow a normal distribution using Shapiro–Wilk tests when parametric tests were performed. Wilcoxon Signed-Rank tests were used to assess significant differences between paired data; when pairing was not possible, data were assessed using Mann–Whitney tests. To determine if there were statistically significant differences between 2 or more groups, one-way ANOVA (Kruskal–Wallis) tests were performed. For proven normally distributed data, ordinary one-way ANOVA and unpaired t-tests were used. A *p* value of <0.05 was deemed statistically significant. Details on the statistical tests used are provided in the figure legends.

## 3. Results

### 3.1. Aerobic Exercise and Resistance Training Is Feasible in a Surgical Pancreatic Cancer Mouse Model

Six–eight-week-old *C57BL/6* mice underwent surgical anaesthesia, laparotomy and orthotopic implantation of PANC02-luc cells. The mice recovered well from the procedure and were able to run 15 min/day at a speed of 12 m/min starting on day 2 after surgery. The mice were also able to hang upside down on an inverted horizontal wire screen for 1 min/twice a week starting on day 2 after surgery. Although growth of the pancreatic tumour was possible to track in certain mice using IVIS, not all tumours were visible. At end of study, the mice were humanely killed and all mice were found to have developed tumours, which were then harvested for flow cytometry and histological analysis. By week 4, 7 out of the 12 mice had visible bioluminescent signals in tumours using the IVIS. However, all mice had tumours on dissection (see Supplementary Figure S1).

### 3.2. Aerobic Exercise and Resistance Training Reduced the Metastatic Potential of Pancreatic Cancer

**Figure 2:**
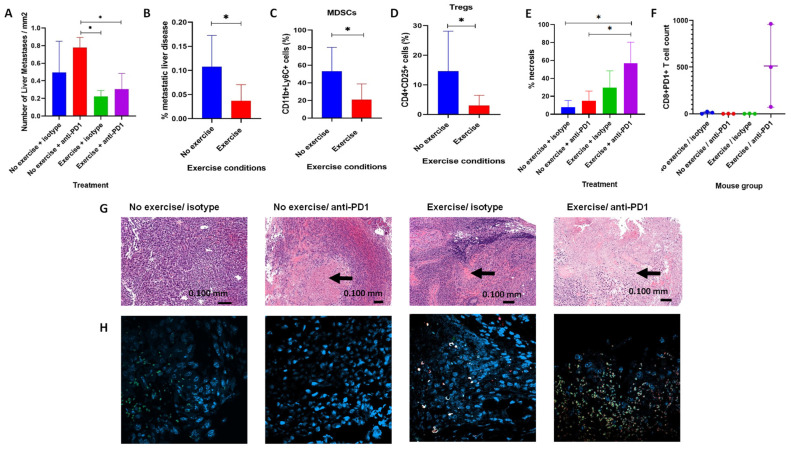
(**A**–**H**) Exercise reduces metastases, modulates the TME and enhances treatment response in combination with anti-PD1 in a pancreatic cancer mouse model. A total of 20 *C57Bl/6* mice underwent orthotopic pancreatic cancer implantation with luciferase-labelled Panc-02 cells and began exercising starting on day 2. Due to surgical complications at the start of the study, 1 mouse from each of the isotype drug groups required humane killing prior to data collection. At the end of study, the tumours were divided in two: one half was prepared for flow cytometry analysis and the other half, along with livers, was fixed in 4% paraformaldehyde, then placed in 70% ethanol, embedded in wax, sectioned and stained with H&E. (**A**) Number of liver metastases per mm^2^ of liver for each subgroup (n = 16). (**B**) The % area of liver affected by micrometastatic disease in non-exercising and exercising cohorts (n = 16). (**C**) MDSCs and (**D**) Tregs within the tumours of non-exercising and exercising cohorts, as determined by flow cytometry (n = 16). (**E**) Percentage of necrosis within the tumours (n = 12). (**F**) ImageJ was used to determine the number of CD8+PD1+ T cells within the different tumours (n = 12). (**G**) SlideViewer was used to image H&E slides of tumours and measure areas of necrosis. (**H**) Slides were de-waxed, stained with immunofluorescent CD8 and PD1 antibodies along with DAPI, and visualised using a Zeiss LSM 980 Airyscan Confocal Microscope (Zeiss, Germany). Kruskal–Wallis test was used as a one-way ANOVA to assess statistical difference between more than 2 groups. Mann–Whitney-U tests were used to determine the statistical significance between two groups ((**A**–**D**,**F**)). Shapiro–Wilk tests were used to determine normality, and if deemed a normal distribution, unpaired T tests were used (**E**). *p* < 0.05 = *.

To evaluate the systemic effects of exercise on cancer cell migration in vivo, the metastatic burden within the liver was measured. In the pilot study, comparing all mice who underwent treadmill running versus no running, a 92.9% reduction in liver metastatic disease was seen in the treadmill cohorts (*p* = 0.0087, Supplementary Figure S2). There were significant reductions in the number of liver micrometastases in both the exercise/isotype and exercise/anti-PD1 groups compared to the no-exercise/anti-PD1 group (*p* = 0.0286, Figure 2A). This significance was strengthened when analysing the exercising cohorts together compared to the non-exercising groups (*p* = 0.0059). To further analyse the nature of these micrometastases, the area of metastatic disease was measured and compared to liver size. The size reduction was significant when the exercising cohorts were compared to the non-exercising groups (*p* = 0.0205, Figure 2B). Amongst the mice that underwent exercise, there was no statistically significant difference in liver micrometastases between the isotype and anti-PD1 treatments.

### 3.3. Aerobic Exercise and Resistance Training Modulate the Pancreatic TME Towards a Hot Climate

Exercise systemically increased NK cells and reduced CD4+CD25+ T cells, which may indicate a reduction in Tregs. Flow cytometry was used to determine if there are any differences in the tumours of the pancreatic cancer mouse model, with Supplementary Figure S3 showing the gating strategy for the mouse immune cell panel. TME analyses from the pancreatic cancer anti-PD1 study demonstrated a significant reduction in MDSCs (73.5%, *p* = 0.0426) (Figure 2C) and Tregs (89.0%, *p* = 0.0115) (Figure 2D) in the exercising groups. No statistically significant differences were observed between the isotype and anti-PD1 treatments. Although not significant, a trend towards a reduction in CD4+CD25+ Tregs and an increase in NK cells within the tumour microenvironment were seen in the exercising groups in the pilot study (*p* = 0.1000 and *p* = 0.3727, respectively, Supplementary Figure S4). These trends were seen in mice that underwent treadmill running and/or horizontal screen resistance training.

### 3.4. Exercise in Combination with Anti-PD1 Enhances Pancreatic Tumour Responses with Increased Tumour Necrosis

To assess the response to treatment within the tumours, the areas of necrosis were measured to indicate the loss of viable tumour tissue. Figure 2E shows the significant difference in tumour necrosis seen between the groups (*p* = 0.0177); the largest degree of necrosis was seen in tumours of the exercise/anti-PD1 group compared to the no-exercise/anti-PD1 group (*p* = 0.0269) and the no-exercise/anti-PD1 group (*p* = 0.0244). Although significance was not reached, a clear trend was demonstrated that indicated that there was increased tumour necrosis in the exercise/anti-PD1 group compared to the exercise/isotype group. This highlights that the combination of exercise and anti-PD1 treatment can increase the effectiveness of treatment responses.

### 3.5. Exercise in Combination with Anti-PD1 Significantly Increases Intratumoural CD8 T Cell Infiltration and Upregulates PD-1 Expression on T Cells in Pancreatic Cancer

To illustrate the mechanism behind the tumour necrosis, immunofluorescent staining of pancreatic tumours was performed to assess CD8 and PD1 expression (Figure 2F). This demonstrated a significant increase in CD8+ cytotoxic T cell infiltration in the exercise/PD1 group (*p* = 0.0368), specifically around areas of necrosis, which were previously demonstrated to be significantly raised in this group. To complement this, PD1 expression was assessed on CD8+ T cells and shown to be significantly higher in the exercise/anti-PD1 group (*p* = 0.0247). The T cells present in the pancreatic tumours of the no-exercise/isotype group did not express PD1. Conversely, CD8+ T cells were more abundant in the exercise/PD1 group, all of which expressed PD1. This indicates that the combination of exercise/anti-PD1 not only increases cytotoxic T cell infiltration intratumourally, but also enhances PD1 expression on these T cells.

### 3.6. Exercise Reduces Bioluminescent Intensity of Luciferase-Labelled Mesothelioma Tumours In Vivo

**Figure 3:**
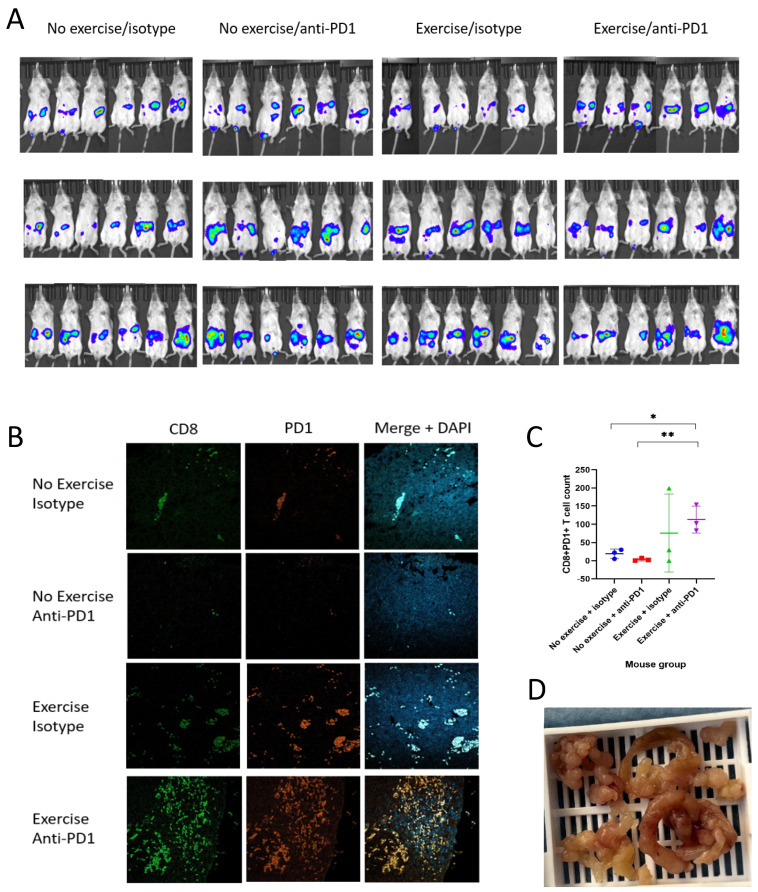
(**A**–**D**) Exercise skews the immune climate towards an anti-cancer phenotype in a mesothelioma mouse model. *BALB/c* mice underwent intraperitoneal mesothelioma implantation with luciferase-labelled AB-1 cells. Mice were categorised into four groups: exercise with anti-PD1, exercise with isotype, no exercise with anti-PD1 and no exercise with isotype. (**A**) IVIS was used to assess the bioluminescence of the tumours. (**B**) Slides were stained with CD8-FITC (green) and PD1-PE (orange) antibodies along with DAPI. A Zeiss LSM 980 Airyscan confocal microscope was used to visualise the slides. (**C**) Significant increases in CD8+PD1+ T cells within the tumours of exercising mice were seen (n = 12). Each dot represents the number of CD8+PD1+ T cells within the tumour of one mouse. (**D**) The blue arrow demonstrates the extensive mesothelioma tumours from a mouse in the no-exercise/isotype group, which shows a ‘string of beads’ appearance. The Kruskal–Wallis test was used as a one-way ANOVA to assess statistical difference between more than 2 groups. Mann–Whitney tests were used to determine statistical significance between two groups. *p* < 0.05 = *, *p* < 0.01 = **.

To assess whether exercise is able to induce similar benefits in another cold tumour, we carried out a mesothelioma mouse study with intraperitoneal implantation of AB-1-luc cells into *BALB/c* mice. Figure 3A illustrates the IVIS imaging of the *BALB/c* mice (n = 24) from weeks 1–3 of the study. Unlike the previous studies, strong bioluminescent intensities were detected in all mice, which corresponded to the presence of mesothelioma tumours in all mice. The mice were able to exercise starting from day 2 following intraperitoneal injection of AB-1 mesothelioma cells. However, the mice were not able to complete 15 min of treadmill running despite modifications including interval running and reducing the speed from 12 m/min to 9 m/min. Therefore, a modified time of 10 min of treadmill running was used for all mice. The mice were able to perform the horizontal screen exercise. 

A trend was seen towards increased bioluminescent radiance in the tumours of the no-exercise/anti-PD1 group and lower radiances in the exercising cohorts, although it was not statistically significant (*p* = 0.42). All mice maintained their weight throughout the study with no significant differences between groups. One mouse in the no-exercise/anti-PD1 group had to be killed one day early on day 15 due to reaching the humane endpoint of poor body condition (reduced mobility and mouse grimace score). All other mice were culled on day 16, with only one of the mice (in the no-exercise/isotype group) reaching the humane endpoint of poor body condition. All mice within the exercise groups maintained good body conditions and mobility.

### 3.7. Exercise Leads to a Trend of a Treg Reduction Within the Mesothelioma TME

To evaluate the changes within the TME, flow cytometry was used to determine immune cell differences in both the effector and immune-tolerant populations. No statistically significant changes were observed in leucocyte subpopulations between mouse groups; however a clear trend was noted: a reduction in Tregs within the exercising cohorts (see Supplemental Materials Figure S4A,B). This supports the Treg reduction seen within pancreatic tumours of exercising mice.

### 3.8. Exercise in Combination with Anti-PD1 Significantly Increases Intratumoural CD8 T Cell Infiltration and Upregulates PD-1 Expression on T Cells in Mesothelioma

Similarly to the pancreatic cancer mouse study, immunofluorescent staining of mesothelioma tumours demonstrated a significant rise in CD8+ cytotoxic T cell infiltration in the exercise/anti-PD1 group compared to the no-exercise/isotype (*p* = 0.0316) and no-exercise/anti-PD1 groups (*p* = 0.0071), as illustrated in Figure 3B. There was a clear upregulation of PD-1 expression along with increased T cell counts within the tumours of the exercise/anti-PD1 group. Figure 3C demonstrates the increase in CD8+PD1+ T cells within the tumours of the exercise/anti-PD1 group compared to the no-exercise /isotype group (*p* = 0.0138) and the no-exercise/anti-PD1 group (*p* = 0.0067). The tumours had a characteristic ‘string of beads’-like appearance, which developed rapidly within the study timeframe, as illustrated in Figure 3D.

### 3.9. Baseline Demographics

A total of 22 healthy volunteers were recruited for the study. The baseline de-mographics are shown in Table 1. Data from both pre- and post-exercise-conditioned blood were available for 20 participants as one participant was excluded due to in-sufficient blood samples and one was excluded due to a technical error with the flow cytometer. The ages of the participants ranged from 23 to 62 years old and included 45% (10) male and 55% (12) female participants, with the majority having no co-morbidities (18, 82%) and 20 were non-smokers (91%). The participants had vary-ing levels of baseline fitness, although all participants performed some form of regular exercise, with 68% (15) performing regular high-intensity exercise during the week.

### 3.10. Exercise Enhances Systemic Anti-Cancer Immunity in Healthy Volunteers

Flow cytometry was used to assess different immune populations in whole blood from the participants before and after an acute high-intensity interval aerobic exercise activity.

**Figure 4:**
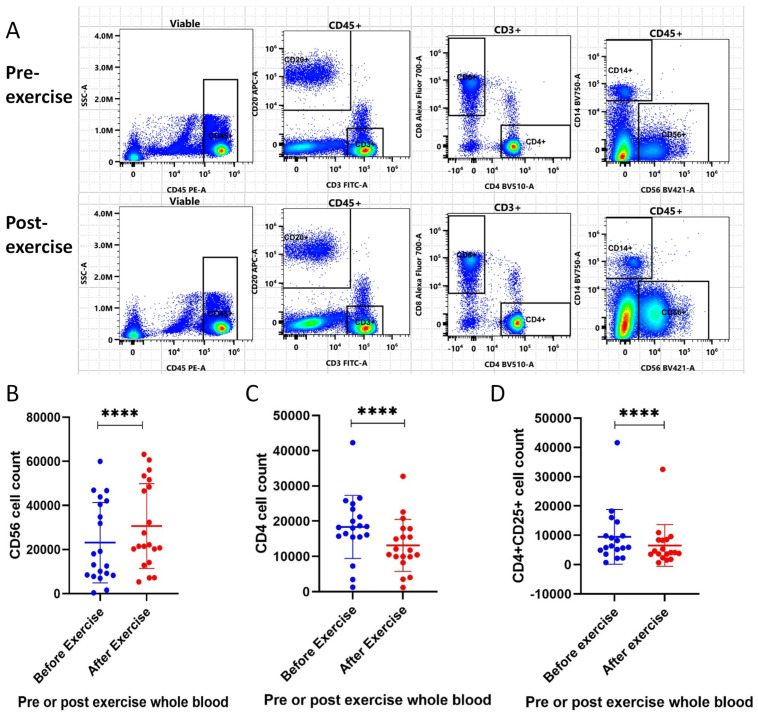
(**A**–**D**) Exercise elicits a systemic immune response that increases NK cells and decreases Tregs. (**A**) Flow cytometry gating strategy comparing pre- and post-exercise-conditioned whole blood from one participant. The top panel illustrates the leucocyte subpopulations within the pre-exercise-conditioned blood of one participant, whilst the bottom panel shows these cells in the blood of the same participant post-exercise. We were unable to perform analysis of CD4+ cell counts on one participant due to lack of availability of the CD4-BV510 conjugate. (**B**–**D**) Scatter plots demonstrating statistically significant differences in (**B**) CD56+ NK (n = 20), (**C**) CD4 (T helper cell) (n = 19) and (**D**) CD4+CD25+ (Treg) cell counts (n = 18) following aerobic exercise. Blood samples were taken prior to and immediately after exercise. Whole-blood samples were stained with CD56-BV421, CD4-BV510 and CD25-PEFire700 prior to red cell lysis and fixation with 1% PFA. Cell counts were quantified using the Cytek Aurora Flow Cytometry System. Data were analysed using GraphPad Prism 8.0.2 with Wilcoxon Signed-Rank tests. *p* < 0.0001 = ****.

Figure 4A illustrates the significant changes seen in CD56+ NK cells before and after the acute high-intensity exercise in one participant. A 32.6% rise in CD56+ NK cells was seen post-aerobic exercise (*p* < 0.0001, 95% CI: 23.0 to 42.2%), as shown in Figure 4B. Interestingly, a significant decrease in CD4 T cells occurred following aerobic exercise (28.5%, *p* < 0.0001, 95% CI: 20.4 to 36.7%) (Figure 4C). Therefore, we investigated if this reduction was seen in the regulatory subset of CD4 T cells as these cells play a role in immune tolerance and are implicated in tumour progression and spread. Furthermore, as shown in Figure 4D, CD4+CD25+ cells (Tregs) were also significantly reduced post-exercise (31.0% reduction, *p* < 0.0001, 95% CI: 13.7–48.3%).

### 3.11. Exercise-Conditioned Serum Delays in Vitro Migration of Pancreatic Cancer Cells

**Figure 5:**
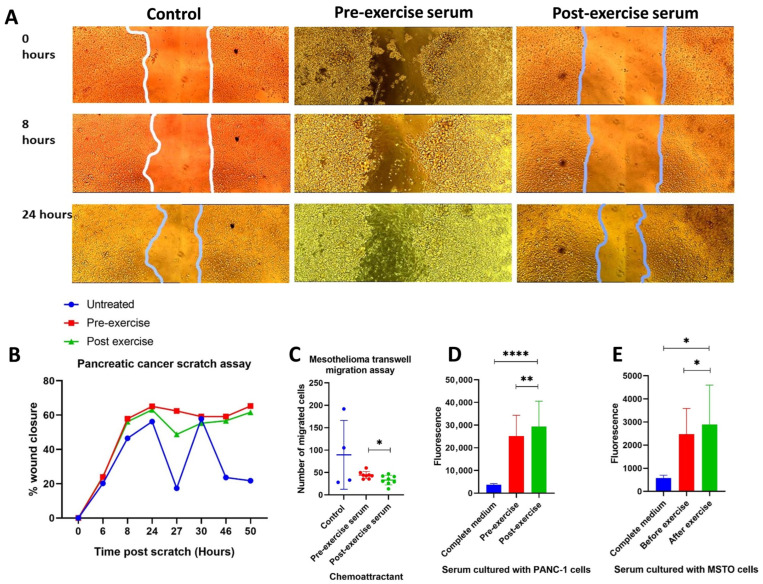
(**A**–**E**) Pancreatic cancer and mesothelioma migration is significantly reduced and apoptosis is increased when cultured with exercise-conditioned serum. (**A**) Scratch assays of PANC-1 cells at 0, 8 and 24 h. Images were taken using the EVOS™ FL Auto Imaging System (ThermoFisher Scientific). (**B**) Summary interleaved bar graph demonstrates percentage wound closure at selected time points between 0 and 50 h after scratching (n = 15). (**C**) Transwell migration assays demonstrated a significant reduction in mesothelioma cell migration with post-exercise-conditioned serum. Differences in cell migration were analysed using GraphPad Prism 8.0.2 with Kruskal–Wallis and Mann–Whitney tests. (**D**) PANC-1 and (**E**) MSTO-211H cells were cultured with either complete medium (10% FBS), or 10% pre-exercise or 10% post-exercise-conditioned serum for 24 h (n = 7). CellEvent™ Caspase-3/7 Red Detection Reagent was added and fluorescence was read using an Ensight Multimode Microplate reader. Significantly increased caspase 3/7 levels were demonstrated when PANC-1 cells and MSTO-211H cells were cultured with post-exercise-conditioned serum compared to pre-exercise-conditioned serum. Wilcoxon Signed-Rank tests were used to determine significance. *p* < 0.05 = *, *p* < 0.01 = **, *p* < 0.0001 ****.

To assess the effects of exercise on pancreatic cancer cell migration, scratch assays were performed with PANC-1 cells cultured in pre- or post-exercise-conditioned serum (Figure 5A).

PANC-1 cells demonstrated delayed wound closure when cultured with exercise-conditioned serum compared to matched pre-exercise-conditioned serum (*p* = 0.0313), as demonstrated in Figure 5B. This indicates that high-intensity aerobic exercise inhibits the migratory potential of pancreatic cancer cells.

### 3.12. Exercise-Conditioned Serum Delays in Vitro Migration of Mesothelioma Cells

Due to difficulties in performing scratch assays with the MSTO-211H cells, transwell migration assays were utilised to assess the migration of mesothelioma cells. A significant reduction in MSTO-211H migration (Figure 5C) was seen with post-exercise-conditioned serum compared to pre-exercise serum (*p* = 0.0156). Supplemental Figure S6 illustrates the immunofluorescent staining of DID-labelled cells captured with the EVOS microscope. The results indicate that mesothelioma migration is inhibited by the effects of high-intensity aerobic exercise.

### 3.13. Exercise-Conditioned Serum Enhances Caspase3/7 Apoptotic Activity in Pancreatic Cancer Cells

Both aerobic and resistance exercise can stimulate myokine release, promoting cancer cell death. To assess this apoptotic effect, caspase-3/7 assays were performed on pancreatic cancer cells cultured with exercise-conditioned serum. Significantly increased caspase 3/7 levels were observed when PANC-1 cells (16.5% increase, *p* = 0.0099) and MSTO-211H cells (6.67% increase, *p* = 0.0313) were cultured with post-exercise-conditioned serum compared to pre-exercise-conditioned serum (Figure 5D,E).

## 4. Discussion

Exercise has been shown to improve survival, quality of life and reduce cancer recurrence in different cancer settings [1,11,12]. Growing evidence supports that aerobic exercise and resistance training promote anti-tumour immunity and may be pivotal in reprogramming cold TMEs to become hot. This study set out to determine the effect of high-intensity interval aerobic exercise on systemic immunity in healthy volunteers and evaluate any exercise-induced changes in cancer cell migration and apoptosis in mesothelioma and pancreatic cancer—tumours at different parts of the cold–hot spectrum. The results then supported the exploration of the effects of a combination of exercise and anti-PD1 therapy in pancreatic cancer and mesothelioma mouse models.

Whilst exercise-induced leucocytosis is an established phenomenon, exercise-induced intratumoural immune effects and the systemic and intratumoural reduction in immune-tolerant cells in pancreatic cancer and mesothelioma are less well described. Our study demonstrates an instantaneous systemic mobilisation of NK cells in all healthy volunteer participants, irrespective of baseline fitness (Supplemental Figure S7).

An abundance of NK cells within the systemic circulation and intratumourally has been correlated with a favourable prognosis and, specifically, a reduced metastatic potential in a range of hot to cold tumours including renal cell carcinoma (hot) and gastric cancer (cold) [13]. Davis et al. correlated improved survival outcomes in patients with pancreatic cancer with higher circulatory NK cell counts [14]. Sottile et al. have shown that NK cell cytotoxicity is effective against mesothelioma cell lines and increased NK cell activity correlated to prolonged overall survival in mesothelioma patients [15]. NK cells are key components of the innate immune system and are usually the initial responders; therefore, as intratumoural NK cell infiltration was only examined at the end of the study, this may account for the reduced numbers noted compared to those for CD8 T cells [16]. Furthermore, NK cells may have played a key protective role in inhibiting/limiting metastases in these models. NCR1 is a receptor involved in NK cell activation and has been shown to be crucial in suppressing metastases in melanoma and lung mouse models when compared to NCR1-deficient mice [17]. Interestingly, primary tumours were unaffected by the lack of NCR1, indicating that the main effect of exercise-induced NK cell systemic release may be to control and eliminate metastatic disease.

Our study highlights the key role that exercise plays in reducing mobilisation of pancreatic cancer cells and limiting metastatic potential. Systemic exercise-induced NK release may be responsible for inhibiting the development and progression of metastases, although the direct mechanism has not been established. Exercise significantly reduced liver micrometastases in this aggressive pancreatic cancer model, irrespective of drug therapy. However, it is unlikely that this suppression of metastases is solely due to NK cell activity. Our in vitro findings showed significantly reduced migration of pancreatic cancer cells when cultured with post-exercise-conditioned serum. Therefore, the presence of anti-migratory factors within exercise serum could play a role in inhibiting metastases. Evidence suggests that exercise-conditioned serum is enriched with myokines, such as irisin [18,19]. Recombinant irisin has been shown to exert anti-migratory properties on pancreatic cancer cells by disrupting the epithelial–mesenchymal transition, preventing detachment from surrounding cells by increasing E-cadherin, and reducing motility by diminishing vimentin [18]. Schwappacher et al. provided evidence supporting the anti-migratory properties of whole-body electromyostimulation (WB-EMS) against pancreatic cancer cells, implicating other myokines such as interleukin (IL)-10 and CCL4 [19]. Future work could include the characterisation of the myokines that could be eliciting these anti-cancer effects. Ideally, serum would be taken at different time points after exercise and within an exercise training programme to fully understand the dynamic changes in myokines that occur with exercise.

CD8+ T cells, effector cells of adaptive immunity, rely on the presentation of tumour antigens on Major Histocompatibility Complex (MHC) class I molecules; thus, NK cells are able to act synergistically to eliminate cancer cells that lack MHC class I expression [20]. Studies have demonstrated favourable outcomes with increased intratumoural CD8+ T cells in both pancreatic cancer and mesothelioma patients [21,22]. A significant increase in CD8 T cell infiltration was demonstrated in the pancreatic tumours of mice receiving anti-PD1 therapy and undergoing regular exercise. These synergistic exercise-induced changes provide excellent immune enhancement in addition to immunotherapy. Furthermore, the upregulation of PD-1 expression on CD8 T cells within the tumours of exercise/anti-PD1 mice may indicate the switch to an active cytotoxic phenotype and highlights the greater target for anti-PD1 treatment; however, further validation would be required to prove CD8 T cell activation, such as interferon gamma (IFN-γ) quantification.

Complementing the increase in CD8 T cells observed, we have demonstrated that a single bout of high-intensity interval aerobic exercise in healthy volunteers reduced systemic Tregs, supporting decrease in Tregs seen in the TMEs of exercising mice in both pancreatic cancer and mesothelioma mouse models. Tregs play an essential role in protecting against autoimmunity by dampening the immune response against self-antigens [23]. However, they support tumour propagation by promoting immune tolerance to tumour-associated antigens [23,24] and have been implicated in disease progression in both pancreatic cancer and mesothelioma [25,26]; therefore, our findings support a promising role of exercise in these patient groups.

TMEs rich in Tregs potentiate a cold phenotype and correlate to immunotherapy resistance and poorer clinical outcomes [24]. Treg-induced immune tolerance is achieved through multiple pathways including high expression of cytotoxic T-lymphocyte-associated protein 4 (CTLA-4), an immune checkpoint protein that binds to CD80/CD86 on antigen-presenting cells to provide inhibitory signals [27]. Tregs are able to manipulate the cytokine profile by depleting IL-2, which is involved in T cell proliferation, as they highly express CD25, which consists of the IL-2 receptor α chain [28]. They release IL-10, IL-35 and Transforming Growth Factor Beta (TGFβ) to create a hostile, ineffective environment for cytotoxic immune cells [28]. Tregs are also directly cytotoxic to CD8+ T and NK cells via the release of perforin and granzyme B (27,28). Furthermore, they possess the chemokine receptors CXCR3, CCR4, CCR8 and CCR10 that allow for intratumoural recruitment of Tregs, potentiating the immunosuppressive TME [28].

Selective depletion of Tregs has been an area of interest to promote anti-tumour activity and potentiate anti-cancer treatment, such as immune checkpoint inhibitors (ICIs). Initial studies had demonstrated that Treg inhibition targeting CD25 exhibits favourable anti-tumour activity and promotion of CD8+ T cell intratumoural infiltration, creating a hot TME [24]. Exercise appears to specifically deplete Tregs within the TMEs of pancreatic cancer, which could be utilised in cancer patients to promote anti-tumour activity, shift TMEs towards the hot end of the spectrum and enhance ICI responses. Future work could include more accurately defining Tregs with FOXP3 staining within the tumours. The changes seen could be confirmed with flow cytometry.

Our study also demonstrated a significant reduction in MDSCs with exercise in the pancreatic cancer model, which further supports the transformation towards a hotter TME and supports the findings from other studies [29].

To our knowledge, our study is the first to demonstrate the benefits of a combined aerobic exercise and resistance training in pancreatic cancer, using less intense aerobic activity of (15 min on a treadmill 4 days/week) compared to previous studies who utilised 30 min of treadmill running 5 days/week [29,30,31]. This is essential to finding the optimal amount of exercise that can provide significant immune benefits whilst also being feasible in an elderly /frail advanced cancer population.

Our study expands upon Kurz et al.’s findings by demonstrating a significant increase in tumour necrosis in the tumours of the exercise/anti-PD1 group, indicating improved tumour responses. This would correlate with improved patient outcomes including survival. Caspase 3/7 assays demonstrated a clear increase in the apoptotic activity of PANC-1 cells when cultured with post-exercise-conditioned serum. Loss of protective apoptotic pathways occur early in the development of pancreatic cancer, and this persistent evasion of apoptosis is partially responsible for anti-cancer treatments being relatively futile [4]. Exercise-mediated apoptosis of tumour cells would be invaluable in improving pancreatic cancer treatments. Our pancreatic cancer mouse studies suggest that combining aerobic exercise and resistance training could boost immunotherapy responsiveness. However, functional validation with CD8 T and NK cell cytotoxicity assays is required to confirm exercise-driven immune-mediated cancer cell death.

Limitations include the use of healthy volunteers who all undertook regular exercise and therefore, the immune changes seen with acute exercise may be modest compared to those who do not regularly exercise, which more accurately reflect the advanced mesothelioma and pancreatic cancer population. Blood tests were done immediately following exercise but at no further time points. Studies have revealed that exercise-induced release of cytokines, such as IL-6, can peak up to 2 h after exercise completion [32]. Therefore, there may not have been enough time to detect a significant change in cytokines, which could account for the lack of cell viability differences between pre- and post-exercise-conditioned sera. Future studies should look at different time points post-exercise, as well as include patients with cancer. Future work should also include exploration of the mechanism behind the observed exercise-induced immune effects using proteomic immune assays to quantify a wide range of cytokines/myokines.

The lack of visibility of the pancreatic tumours with the IVIS suggests issues with regard to luciferase expression as well as potential interference from the black fur on the mice. Reviewing the effect of exercise in a different pancreatic cancer model with a different cell line could provide validation of its beneficial effects.

## 5. Conclusions

In summary, our findings have shown a single bout of high-intensity interval aerobic exercise significantly increases circulating NK cells and reduces Tregs, providing the optimum environment to boost anti-cancer activity. Exercise-conditioned serum exerts anti-cancer effects against pancreatic cancer and mesothelioma cells in vitro. Moderate-intensity exercise combined with anti-PD1 increases tumour necrosis reduces metastatic disease and transforms the TME towards a hot phenotype. This provides a strong basis for investigating whether this effect can be seen systemically and intratumourally in patients with advanced cancer, warranting further exploration with clinical trials.

## Figures and Tables

**Table 1:** Baseline characteristics of the healthy volunteers.

Age (Years)	Number of Participants
23–25	2
26–30	7
31–35	7
36–40	0
41–45	2
46–50	1
51–55	1
56–60	1
61–65	1
Gender	
Male	10
Female	12
Co-morbidities	
0	18
1	2
2 or more	2
Baseline fitness	
No regular exercise	0
Moderate intensity 1–2 times/week	2
Moderate intensity 3–4 times/week	2
Moderate intensity 5–7 times/week	3
High intensity 1–2 times/week	4
High intensity 3–4 times/week	5
High intensity 5–7 times/week	6
Smoker	
Y	2
N	20

## Data Availability

The original contributions presented in this study are included in the article/Supplementary Material. Further inquiries can be directed to the corresponding author(s).

## Supplementary Materials

The following supporting information can be downloaded at: https://www.mdpi.com/article/10.3390/biom16040493/s1, Figure S1: IVIS images of mice from pilot study; Figure S2: Aerobic exercise reduces the number and burden of liver metastases in pancreatic cancer; Figure S3: Flow cytometry gating strategy mouse immune panel; Figure S4: Aerobic exercise and resistance training causes a trend towards favourable anti-cancer tumour immune microenvironment; Figure S5: Aerobic exercise and resistance training causes a trend towards a reduction in Tregs; Figure S6: MSTO-211H cell migration is significantly reduced with exercise conditioned serum; Figure S7: Exercise-induced T helper cell and Treg decreases are dependent on age; Table S1: Panel containing 10 antibody-fluorophore conjugates; Table S2: (A) Experiment details for pilot study. (B) Experiment details for pancreatic cancer/anti-PD1 study. (C) Experiment details for Mesothelioma/anti-PD1 study.

## List of Abbreviations

NHS	National Health Service
WCRF	the World Cancer Research Fund
TME	tumour microenvironment
RPMI	Roswell Park Memorial Institute
FBS	foetal bovine serum
DMEM	Dulbecco’s Modified Eagle Medium
CO_2_	carbon dioxide
s.c.	subcutaneous
i.p.	intraperitoneal
IVIS	In Vivo Imaging System
PD1	anti-Programmed Cell Death Protein 1
EDTA	ethylenediaminetetraacetic acid
RPM	revolutions per minute
PFA	paraformaldehyde
PBS	phosphate-buffered saline
RPE	Rate of Perceived Exertion
Tregs	regulatory T cells
MDSCs	Myeloid-Derived Suppressor Cells
NK	Natural Killer cells
H&E	haematoxylin and eosin
CTLA-4	cytotoxic T-lymphocyte-associated protein 4
WB-EMS	whole-body electromyostimulation
IL	interleukin
MHC	Major Histocompatibility Complex
TGFβ	Transforming Growth Factor Beta
ICIs	immune checkpoint inhibitors
