# Supplementary material for: Exercise Boosts the Immune System and Enhances Immunotherapy Responses in Pancreatic Cancer and Mesothelioma
Source: Biomolecules. 2026 Mar 25;16(4):493. doi: 10.3390/biom16040493 (PMC13113466; doi:10.3390/biom16040493)
Supplement: Supplementary file 1 [file biomolecules-16-00493-s001.zip › biomolecules-4181633-supplementary.pdf]

Supplemental material

Table S1. Panel containing 10 antibody-fluorophore conjugates.

Antibodies	Description	Fluorophores
CD3	General T cell marker	FITC
CD4	T helper cells	BV510
CD8	Cytotoxic T cells	AF700
CD14	Monocytes	BV750
CD20	B cells	APC
CD45	General leucocyte marker	PE
CD56	Natural Killer (NK) cells	BV421
CD25	T cell activation marker	PEFire700
CD69	Early T cell activation marker	BV650
HLA-DR	T cell activation marker	PECy7

Table S2A: Experiment details for pilot study

C57 BL/6 mice		Tumour development			Treatment
Exp group	No. mice	Cells	Route Admin	Volume (μl)	
1	3	4×10^5 luciferase Labelled Panc-02 cells	Pancreatic injection	20ul	Sedentary
2	3				Horizontal screen exercise
3	3				Treadmill
4	3				Horizontal screen exercise and treadmill

Table S2B: Experiment details for pancreatic cancer/anti-PD1 study

C57 BL/6 mice		Tumour development			Treatment		
Exp group	No. mice	Cells	Route Admin	Volume (µl)	Treatment	Route Admin of drug	Volume of drug (µl)
1	5	4×10^5 luciferase labelled Panc-02 cells	Pancreatic injection	20ul	Isotype control + no exercise	I.P.	100ul
2	5				Isotype control + exercise		
3	5				Anti-PD1 + no exercise		
4	5				Anti-PD1 + exercise		

Table S2C: Experiment details for Mesothelioma/anti-PD1 study

C57 BL/6 mice		Tumour development			Treatment		
Exp group	No. mice	Cells	Route Admin	Volume (µl)	Treatment	Route Admin of drug	Volume of drug (µl)
1	6	1×10^5 luciferase labelled AB-1 cells	I.P.	100ul	Isotype control + no exercise	I.P.	100ul
2	6				Isotype control + exercise		

3	6				Anti-PD1 + no exercise		
4	6				Anti-PD1 + exercise		

Supplemental material

Pilot study data

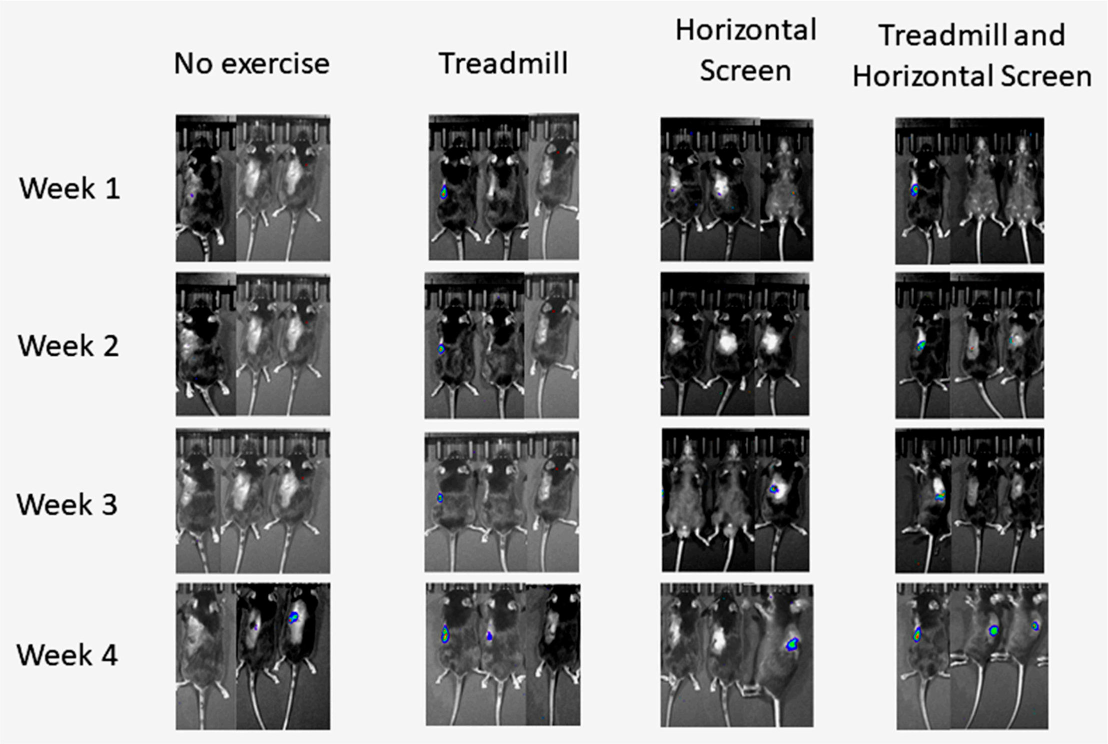

Figure S1: IVIS images of mice from pilot study

C57Bl/6 mice underwent orthotopic pancreatic cancer implantation with $4x10^5$ luciferase labelled Panc-02 cells. Mice were categorised into four groups: no exercise, treadmill only 4 days/week, horizontal screen resistance training 2 days/week and a combination of treadmill and horizontal screen training. IVIS was used to assess bioluminescence of the tumour weekly illustrated. This figure demonstrates the 12 C57BL/6 mice in the pilot pancreatic cancer study from weeks 1-4. By week 4, 7 out of the 12 mice had visible bioluminescent signalling from tumours with IVIS. However, all mice had tumours on dissection. All mice maintained weight and good condition throughout the study

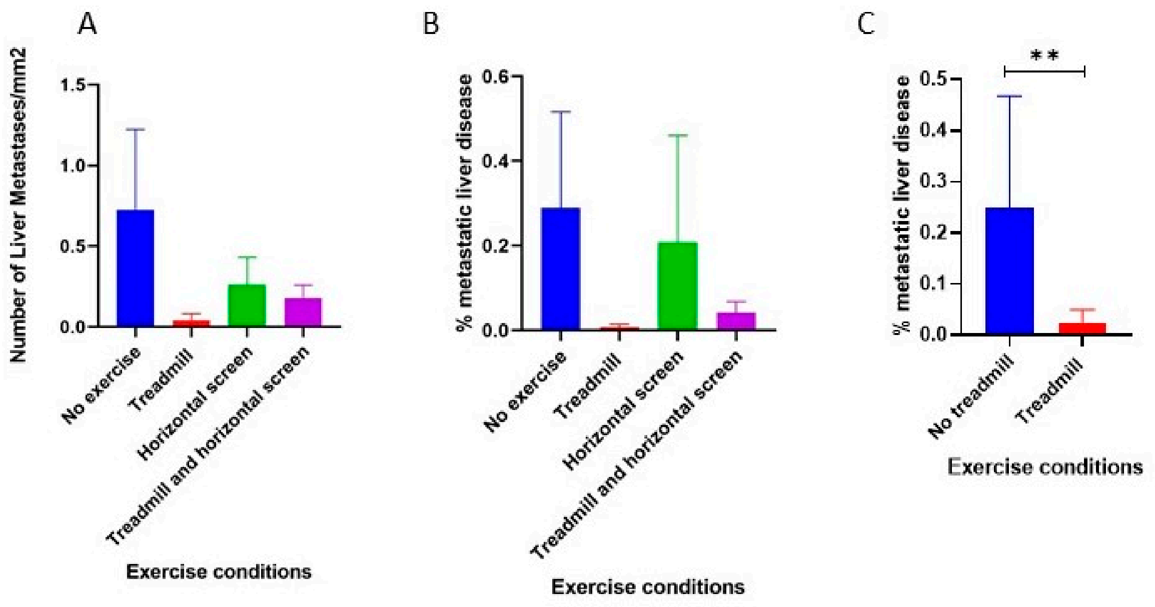

Figure S2A-C: Aerobic exercise reduces the number and burden of liver metastases in pancreatic cancer

One liver lobe from each mouse was analysed using SlideViewer, areas of metastatic disease were counted and measured comparatively to the area of the liver lobe to calculate the number of liver metastases and the % of liver affected by metastatic disease. Kruskal-Wallis test was used as a one-way ANOVA to assess statistical difference between more than 2 groups. Mann-Whitney-U tests were used to ascertain statistical significance between two groups. A statistically significant difference in the **A.** number and **B.** area of liver metastases was seen between groups (p=0.0382 and p=0.0179 respectively). **C.** Comparing all mice who underwent treadmill running versus no running, a 92.9% reduction in % metastatic area was seen in the treadmill cohorts (p=0.0087) ** p<0.005, * p<0.05

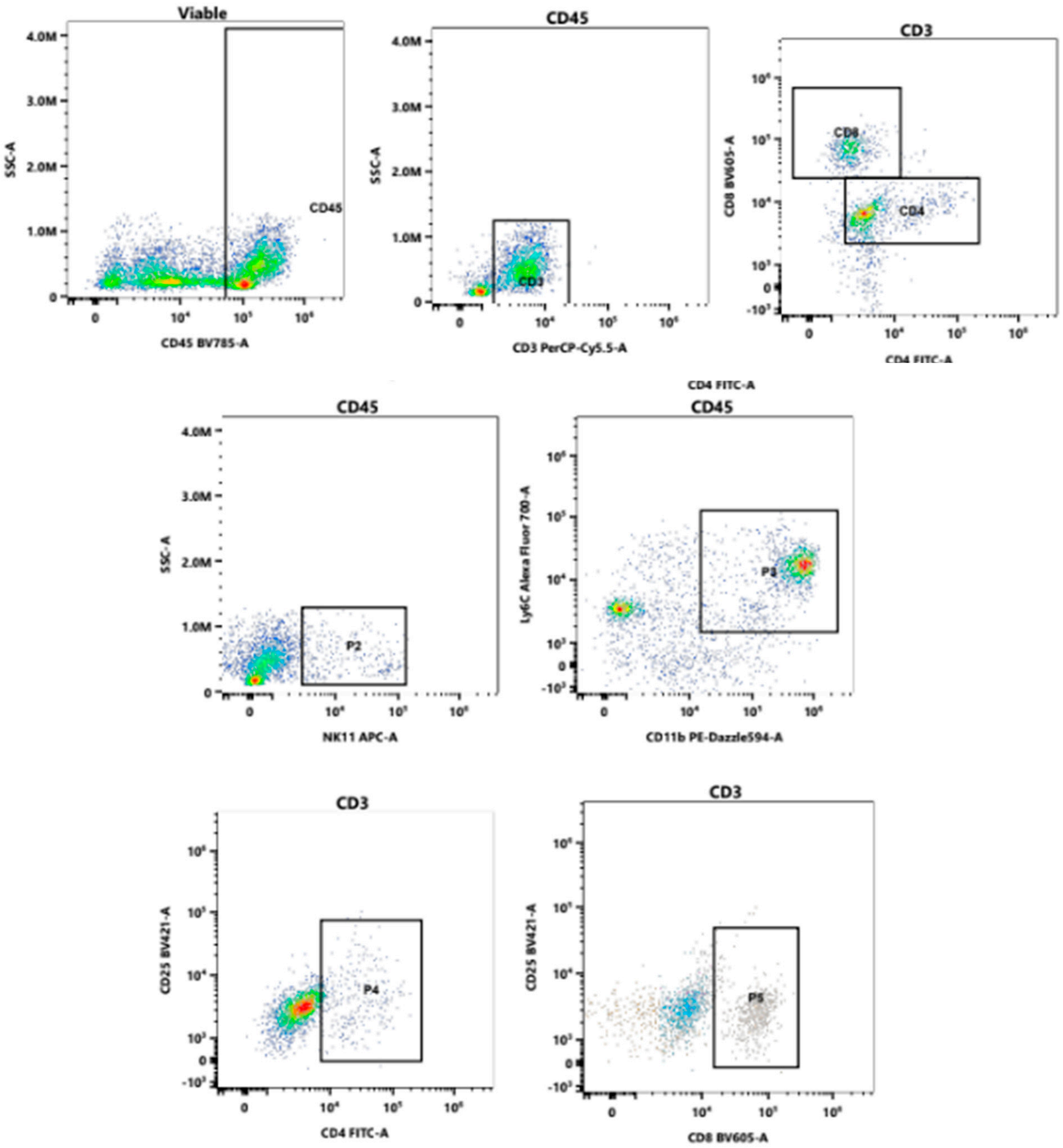

Figure S3A-G: Flow cytometry gating strategy mouse immune panel.

Representative dot plots of viable A. CD45 (general leucocyte marker) cells were selected using the Cytek Aurora Flow Cytometry System and separated into B. CD3+ cells (T cells). C. The CD3+ T cell population was used to identify CD4+ (T helper) and CD8+ (cytotoxic) T cells. D. NK1.1 cells (NK cells) and CD11b+Ly6C cells (Myeloid Derived Suppressor Cells) were gated from viable CD45 populations. F-G. CD25, T cell activation marker, was measured within the CD4+ and CD8+ T cell populations.

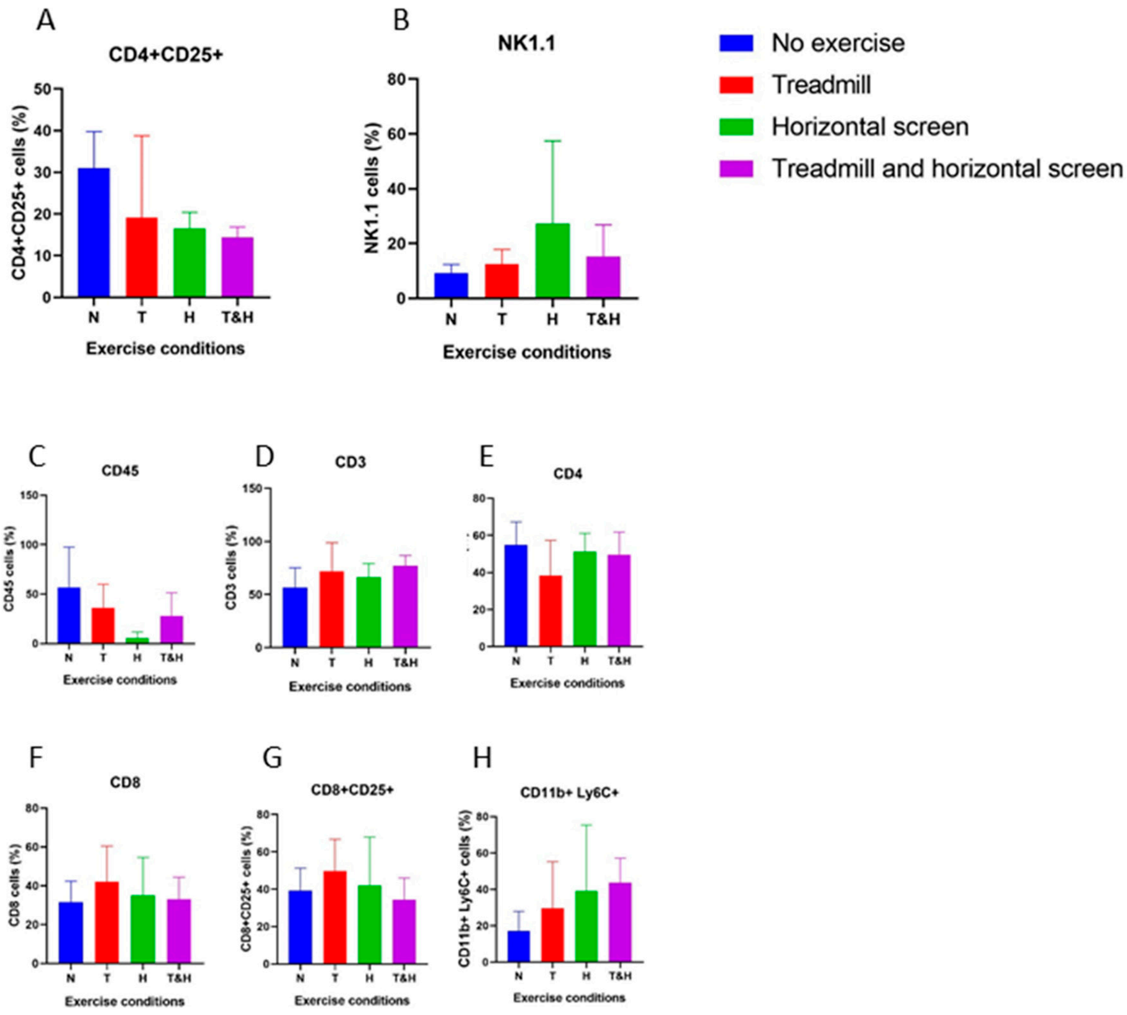

Figure S4A-H: Aerobic exercise and resistance training causes a trend towards favourable anti-cancer tumour immune microenvironment

This flow cytometry immune panel assessed A. CD45, B. CD3, C. CD3+CD4+, D. CD3+CD4+CD25+, E. CD3+CD8+, F. CD3+CD8+CD25+, G. NK1.1 and H. CD11b+Ly6C+ within the pancreatic tumours on completion of the study. Trends towards reduced CD4+CD25+ Tregs and increase in activated CD8+CD25+ cytotoxic T cells were seen with treadmill and horizontal screen training. A trend towards raised NK1.1 cells was seen with horizontal screen and treadmill training. 2-way ANOVA and Mann-Whitney-U tests were used to ascertain statistical significance, which was not reached.

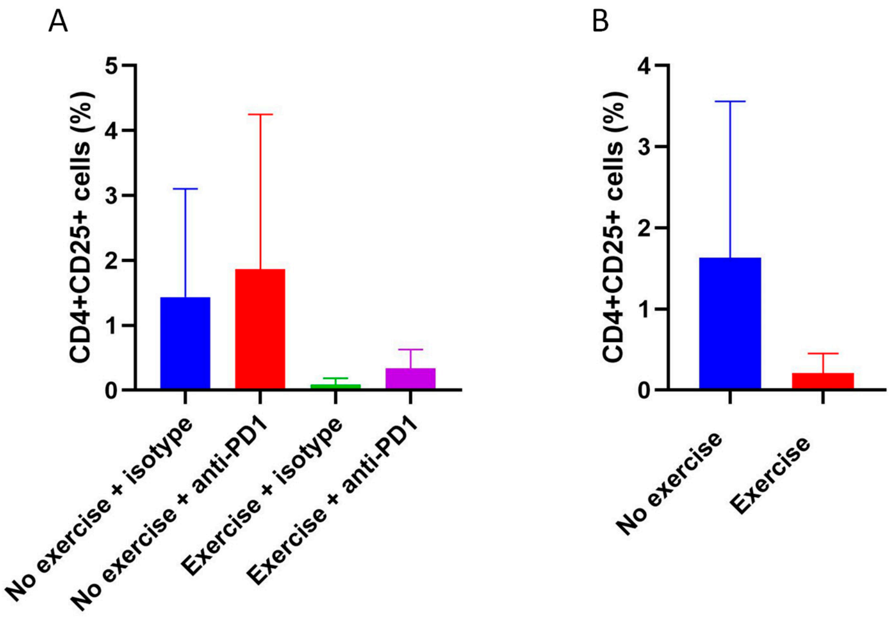

Figure S5A-B: Aerobic exercise and resistance training causes a trend towards a reduction in Tregs

Flow cytometry was used to assess an immune panel including CD45, CD3, CD4 and CD25 on the mesothelioma tumours on completion of the study. **A.** A trend towards reduced CD4+CD25+ Tregs was seen with exercise and anti-PD1 and exercise and isotype. **B.** A clear trend is demonstrated towards a Tregs reduction in the exercising cohorts. One-way ANOVA and Mann-Whitney tests were used to ascertain statistical significance, which was not reached.

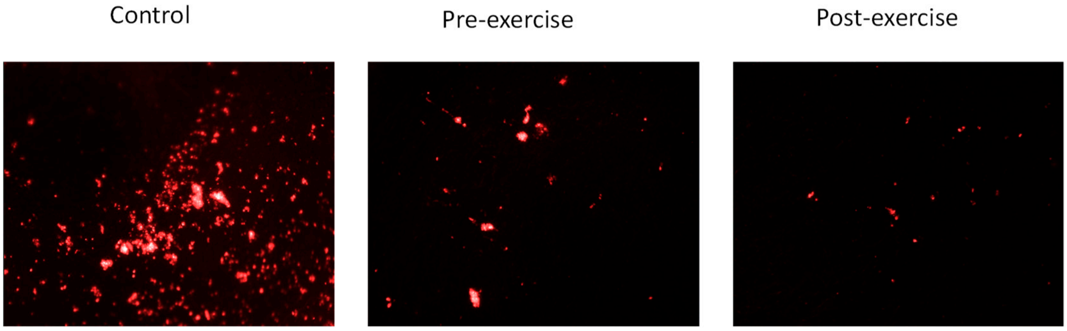

Figure S6: MSTO-211H cell migration is significantly reduced with exercise conditioned serum

Transwell migration assay of MSTO-211H mesothelioma cells. $1x10^5$ MSTO-211H cells in 100µl of complete RPMI medium stained with DID were seeded on 3.0µm pore cell culture inserts and placed in a 24 well plate. After 10 minutes of incubation at 37°C, wells for filled with 600µl of complete medium (control), or 540µl of serum-free medium with 60µl of pre- or post-exercise conditioned serum (n=8). Plates were incubated for 2 hours, inserts removed, and cotton-tipped applicators used to remove non-migrated sides from apical membrane. Migrated cells on the basal surface of the membrane were fixed using 4% PFA. Images of basal membrane taken using EVOS™ FL Auto Imaging System (ThermoFisher Scientific).

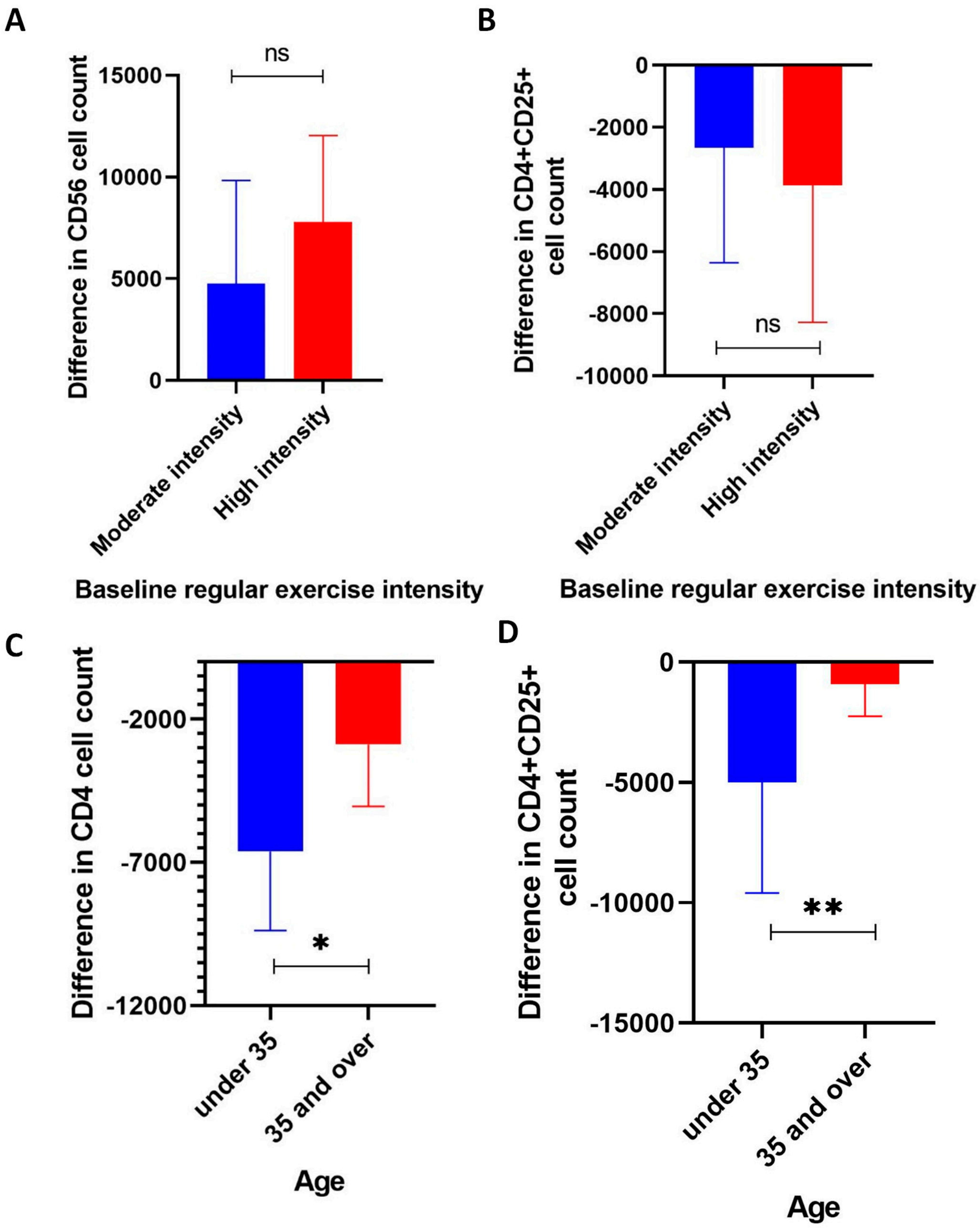

Figure S7A-D: Exercise-induced T helper cell and Treg decreases are dependent on age

Column bar graphs demonstrating exercise-induced changes in **A.** NK cells (CD56) depending on baseline fitness and changes in **B.** T regulatory cells (CD4+CD25+ T cells) depending on baseline fitness. Fast walking, jogging and cycling at a gentle pace were defined as moderate intensity, whilst running, cycling at a fast pace and intense gym exercises were classed as high intensity. **C.** Illustrates the differences seen in exercise-induced T helper cell (CD4) reduction depending on age. **D.** Illustrates T regulatory cell (CD4+CD25+ T cells) decreases depending on age. Cell counts were quantified using the Cytek Aurora Flow Cytometry System and data were analysed with Mann-Whitney tests using GraphPad Prism 8.0.2.
